# Bias and balance in psychedelic academia: a tricky business

**DOI:** 10.47626/2237-6089-2024-0990

**Published:** 2025-09-09

**Authors:** Jordan Bawks, Fabiano A. Gomes

**Affiliations:** 1 McMaster University Department of Psychiatry and Behavioural Neurosciences Hamilton ON Canada Department of Psychiatry and Behavioural Neurosciences, McMaster University, Hamilton, ON, Canada.

Psychedelic assisted psychotherapy is a promising emerging treatment strategy for several psychiatric conditions. Over the last 15 years interest in this approach has exploded among the clinicians, academics and the lay public. The analogy of a gold fever or rush is apt.

It is striking to see how quickly things have moved in all spheres. In the modern era of clinical research, the first safety and efficacy study of a serotonergic psychedelic for a clinical population was a small psilocybin trial for obsessive-compulsive disorder (OCD) published in 2006.^1^ Psilocybin trials quickly moved to other conditions; end of life anxiety in 2011,^2^ tobacco dependence in 2014,^3^ alcohol dependence in 2015,^4^ and depression in 2016.^5^ Other psychedelics quickly followed too, MDMA for post-traumatic stress disorder (PTSD) in 2011^6^ and LSD for end of life anxiety in 2014.^7^ The investigative journalist Michael Pollan brought the topic to public attention with his book *How to Change Your Mind* in 2018, which was then made into a Netflix documentary in 2022. By 2024, Yao et al.^8^ identified 126 trials spanning the 4 major serotonergic psychedelics; MDMA, LSD, psilocybin and ayahuasca.

It is easy for clinicians, patients and healthcare stakeholders to see the tremendous value of a focused intervention that could put mental health symptoms into remission. This is a rare resource, essentially one-of-a-kind in the mental health world. For researchers and academics, they were presented with several different psychedelic compounds to study, and a dozen or more psychiatric conditions to test them on. The rush was, and still is, on.

Excitement must be tempered by caution and reflection so that patient safety and process integrity remain the foundation of clinical research. Concerns around these issues were central in the US Food and Drug Administrations decision to vote against the approval of MDMA psychotherapy for PTSD despite the submitted data demonstrating 80% of patients showing significant improvements.^9^ This is why this article by Koning, Solmi and Brietzke^10^ is so important. The authors investigate sources of bias in safety outcome reporting and the implications for scientific communication. They advocate for increased ownership of biases, minimizing conflicts of interest wherever possible, firmer establishment of safety metrics and increased balance in scientific communication.

Conceptually, this call for increased self-awareness and thoughtful, balanced communication in psychedelic academia is laudable. In practice, we are immediately confronted by the dialectical nature of these demands and see why they are so hard to uphold. While the article advocates for ownership of bias, and for reflection on balanced communication in scientific writing, it never explicitly subjects itself to self-examination on these fronts. As an ordinary example, while the "declaration of interest" section is dutifully filled out, there is no commentary on personal involvement in psychedelics, psychedelic drug research, clinical practice involving any of the medications in question or any acknowledgement of the benefits of publication. With respect to these issues of bias and transparency, even writing this editorial, the present authors note how unnatural and hard to integrate these kinds of self-disclosures and commentary are given the space constraints and typical frame of scientific writing.

Providing a totally balanced scientific communication is such a challenge that one could argue that there are some sections in the paper where an untrained reader could easily be left with an incomplete picture of the scientific landscape.

For instance, the grouping of ketamine, MDMA and the 5HT-2a agonists under the collective umbrella of psychedelics. There are reasonable objections about this premise on several fronts, but perhaps the most relevant one to the theme of responsible scientific communication is that it is risks propagating the misunderstanding among the public that these drugs are equivalent. These drugs have very different pharmacodynamic, safety, efficacy and psychophysiological profiles. Arguably, this misinformation is already being exploited by private ketamine clinics leveraging public awareness and interest in psychedelic psychotherapy by offering "Ketamine Assisted Psychotherapy (KAP)" and or ketamine under the guise of "Psychedelic assisted Psychotherapy." This is a quickly growing and poorly regulated industry in Canda and the United States, with a 2024 NPR article finding between 500-750 active ketamine clinics in the United States.^11^ At the time of writing this article, a quick google search reveals at least 5 such clinics in the "Greater Toronto Area" accessible locally to the authors by self-referral. While there is excellent evidence for ketamine as a treatment for treatment-resistant depression (TRD),^12^ there are very few controlled studies of ketamine assisted psychotherapy, and the only controlled study in KAP for depression is negative.^13^

From the authors standpoint, ketamine is helpful to include under the umbrella of psychedelics so that it can be subject to investigation in the article. However, doing so without adequate differentiation from the other medications mentioned in the article risks the exact propagation of misinformation they are trying to combat. How much differentiation would be required to mitigate this risk? How much would it actively confuse readers and detract from the other salient points the authors are trying to make throughout the article?

To demonstrate this, let us try and provide a counterbalance to our assertion about the weakness of KAP data. After all, we are sure there are many clinicians, providers and patients who would take issue with it. In-fact, if we look at the conclusions of the original randomized clinical trial of KAP for depression, instead of looking at the later meta-analysis, we will see the authors themselves take a different stance on their findings "This proof-of-concept study provides preliminary data indicating that cognitive behavioural therapy (CBT) may sustain the antidepressant effects of ketamine in TRD."^14^ There is also a 2022 systematic review of KAP that suggested "psychotherapy provided before, during and following ketamine sessions, can maximize and prolong benefits [for pain, anxiety and depressive symptoms]."^15^

We now have established two scientific points of view on KAP in dialectical tension. To attempt to resolve these would force us to examine the methods, risks of bias and data that are used by these various authors of these articles to make their claims. Astute readers will notice that we have come full circle to the very things Koning, Solmi and Brietzke^10^ are advocating for to help us find clarity in this exciting world of psychedelic psychotherapy. Along the way, we hope we have highlighted how complicated these issues of biases and balance are, not just in the realm of psychedelics, but in the scientific endeavour as a whole.

## Data Availability

Not applicable.

## References

[1] Moreno FA, Wiegand CB, Taitano EK, Delgado PL (2006). Safety, tolerability, and efficacy of psilocybin in 9 patients with obsessive-compulsive disorder. J Clin Psychiatry.

[2] Grob CS, Danforth AL, Chopra GS, Hagerty M, McKay CR, Halberstadt AL (2011). Pilot study of psilocybin treatment for anxiety in patients with advanced-stage cancer. Arch Gen Psychiatry.

[3] Johnson MW, Garcia-Romeu A, Cosimano MP, Griffiths RR (2014). Pilot study of the 5-HT2AR agonist psilocybin in the treatment of tobacco addiction. J Psychopharmacol.

[4] Bogenschutz MP, Forcehimes AA, Pommy JA, Wilcox CE, Barbosa PC, Strassman RJ (2015). Psilocybin-assisted treatment for alcohol dependence: a proof-of-concept study. J Psychopharmacol.

[5] Carhart-Harris RL, Bolstridge M, Rucker J, Day CM, Erritzoe D, Kaelen M, Bloomfield M, Rickard JA, Forbes B, Feilding A, Taylor D, Pilling S, Curran VH, Nutt DJ (2016). Psilocybin with psychological support for treatment-resistant depression: an open-label feasibility study. Lancet Psychiatry.

[6] Mithoefer MC, Wagner MT, Mithoefer AT, Jerome L, Doblin R (2011). The safety and efficacy of {+/-}3,4-methylenedioxymethamphetamine-assisted psychotherapy in subjects with chronic, treatment-resistant posttraumatic stress disorder: the first randomized controlled pilot study. J Psychopharmacol.

[7] Gasser P, Holstein D, Michel Y, Doblin R, Yazar-Klosinski B, Passie T, Brenneisen R (2014). Safety and efficacy of lysergic acid diethylamide-assisted psychotherapy for anxiety associated with life-threatening diseases. J Nerv Ment Dis.

[8] Yao Y, Guo D, Lu TS, Liu FL, Huang SH, Diao MQ, Li SX, Zhang XJ, Kosten TR, Shi J, Bao YP, Lu L, Han Y (2024). Efficacy and safety of psychedelics for the treatment of mental disorders: A systematic review and meta-analysis. Psychiatry Res.

[9] https://www.nature.com/articles/d41586-024-01622-3.

[10] Koning E, Solmi M, Brietzke E (2025). The influence of stakeholder interests on safety outcome reporting in psychedelic research and implications for science communication. Trends Psychiatry Psychother.

[11] https://www.npr.org/sections/health-shots/2024/01/30/1227630630/ketamine-infusion-clinic-mental-health-depression-anxiety-fda-off-label.

[12] Seshadri A, Prokop LJ, Singh B (2024). Efficacy of intravenous ketamine and intranasal esketamine with dose escalation for Major depression: A systematic review and meta-analysis. J Affect Disord.

[13] Joneborg I, Lee Y, Di Vincenzo JD, Ceban F, Meshkat S, Lui LMW (2022). Active mechanisms of ketamine-assisted psychotherapy: A systematic review. J Affect Disord.

[14] Wilkinson ST, Rhee TG, Joormann J, Webler R, Ortiz Lopez M, Kitay B (2021). Cognitive Behavioral Therapy to Sustain the Antidepressant Effects of Ketamine in Treatment-Resistant Depression: A Randomized Clinical Trial. Psychother Psychosom.

[15] Drozdz SJ, Goel A, Mcgarr MW, Katz J, Ritvo P, Mattina GF (2022). Ketamine Assisted Psychotherapy: A Systematic Narrative Review of the Literature. J Pain Res.

